# The HSP90 Inhibitor, AUY-922, Ameliorates the Development of Nitrogen Mustard-Induced Pulmonary Fibrosis and Lung Dysfunction in Mice

**DOI:** 10.3390/ijms21134740

**Published:** 2020-07-03

**Authors:** Pavel Solopov, Ruben M. L. Colunga Biancatelli, Margarita Marinova, Christiana Dimitropoulou, John D. Catravas

**Affiliations:** 1 Frank Reidy Research Center for Bioelectrics, Old Dominion University, Norfolk, VA 23508, USA; psolopov@odu.edu (P.S.); rcolunga@odu.edu (R.M.L.C.B.); m.d.marinova@gmail.com (M.M.); cdimitro@odu.edu (C.D.); 2 Policlinico Umberto I, La Sapienza University of Rome, 00185 Rome, Italy; 3 School of Medical Diagnostic & Translational Sciences, College of Health Sciences, Old Dominion University, Norfolk, VA 23508, USA

**Keywords:** nitrogen mustard, acute lung injury, pulmonary fibrosis, P-HSP90, ERK, HSP90 inhibitors, anti-fibrotic, AUY-922

## Abstract

Increased levels of heat shock protein 90 (HSP90) have been recently implicated in the pathogenesis of pulmonary fibrosis and the use of HSP90 inhibitors constitutes a potential therapeutic approach. Similarly, acute exposure to nitrogen mustard (NM) is related to the development of chronic lung injury driven by TNF-α, TGF-β, ERK and HSP90. Thus, we developed a murine model of NM-induced pulmonary fibrosis by instilling C57BI/6J mice with 0.625 mg/kg mechlorethamine hydrochloride. After 24 h, mice began receiving AUY-922, a second generation HSP90 inhibitor, at 1 mg/kg 2 times per week or 2 mg/kg 3 times per week, for either 10 or 30 days. AUY-922 suppressed the NM-induced sustained inflammation, as reflected in the reduction of leukocyte and protein concentrations in bronchoalveolar lavage fluid (BALF), and inhibited the activation of pro-fibrotic biomarkers, ERK and HSP90. Furthermore, AUY-922 maintained normal lung function, decreased the overexpression and accumulation of extracellular matrix proteins, and dramatically reduced histologic evidence of fibrosis in the lungs of mice exposed to NM. The HSP90 inhibitor, AUY-922, successfully blocked the adverse effects associated with acute exposures to NM, representing a promising approach against NM-induced pulmonary fibrosis.

## 1. Introduction

Nitrogen mustards (NMs) are cytotoxic agents derived from mustard gas that has been used as chemical warfare in wars of the 20th century. Mustard gas initially causes dermatologic and respiratory toxicity, but once absorbed can provoke neurologic, gastrointestinal, or systemic complications. Developed during World War I, Bis-(2-chloroethyl) methylamine, the first generation of NM, has never been employed in war, and instead has found its application as a chemotherapeutic drug for the treatment of cancer [1,2]. NM inhalation, however, causes both acute and chronic respiratory toxic effects, which appear with a sparse symptomatology of chest tightness, hacking cough and rhinorrhea, but may lead to bronchiolitis, alveolar emphysema and pulmonary fibrosis [3,4,5]. NM is capable of producing a persistent damage through DNA alkylation in guanine N-7 [6], DNA strand breaks, activation of DNA mediated apoptotic pathways, the release of pro-inflammatory cytokines such as TNF-α, IL-1, IL-6, IL-8 and inducible nitric oxide synthase (iNOS) [7,8]. These changes result in nitrosative stress and the overproduction of reactive nitrogen species (RNS), which, in patients with interstitial lung disease (ILD), has been implicated in the pathogenesis of the alveolar damage [9] and has been shown to be a promising biomarker for disease diagnosis and progression [10].

Currently, there are no approved therapies able to treat successfully either acute or chronic lung injuries and prevent the degeneration of lung parenchyma to pulmonary fibrosis, after nitrogen mustard exposure. Laser therapy and the application of airway stents have limited effectiveness [11] and besides inhaled corticosteroids and β2-adrenergic agonists thathave shown partial benefit in improving acute symptomatology, there is no treatment capable of preventing the long-term complications [12].

Recently, it was reported that high levels of heat shock protein 90 (HSP90) are associated with the development of lung fibrosis in patients diagnosed with idiopathic pulmonary fibrosis (IPF) [13]. This protein is an ATP-dependent chaperone, part of the heat shock protein family, which plays a critical role in guaranteeing cellular homeostasis after exposure to environmental and physiological stressors [14]. HSPs, including HSP90, greatly contribute to key characteristic features of cancerous cells, such as uncontrolled proliferation, tissue invasion and promotion of angiogenesis. Moreover, HSP90 facilitates the survival of many oncogenic proteins and therefore has become a valuable target for cancer therapies [15]. Recent studies have suggested that HSP90 inhibitors could be used as a potentially effective treatment for patients with pulmonary fibrosis [13,16,17].

We recently reported that a single exposure of mice to mechlorethamine hydrochloride, induces chronic lung injury and pulmonary fibrosis, thus representing a suitable animal model to study the long-term effects of NM on the lower respiratory tract and its possible countermeasures [18]. Ten days after intratracheal instillation of NM, mice exhibit progressive deposition of collagen and extracellular matrix, which correlates with HSP90 activation (P-HSP90) and culminates at 30 days post-exposure in an overall aberrant destruction of parenchymal architecture and scar tissue formation. The aim of this study is to test the hypothesis that inhibition of HSP90 by AUY-922, could represent a new pharmacological approach against chemically induced pulmonary fibrosis.

## 2. Results

### 2.1. AUY-922 Reduces NM-Induced Weight Loss

**Figure 1:**
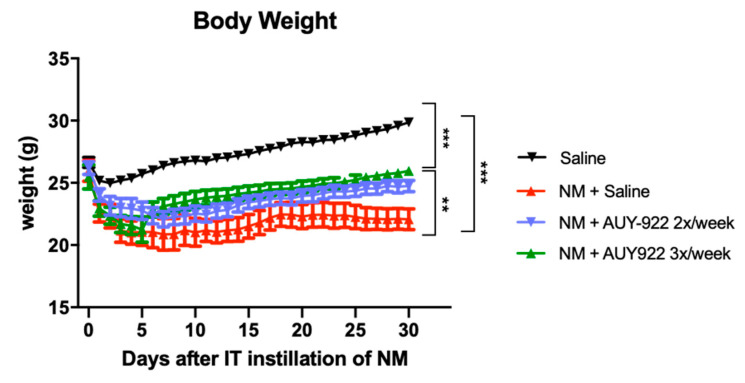
Body weight changes in mice after intratracheal instillation of 0.625 mg/kg nitrogen mustard (NM) or saline and treatment with AUY-922; ***: *p* < 0.001, **: *p* < 0.01 with ANOVA and Turkey’s. *n* = 6 mice per group.

A decrease in body weight was noted the day after instillation of either nitrogen mustard or saline. Mice treated with saline, recovered quickly, and resumed normal weight gain (Figure 1). The decrease in body weight in mice instilled with NM was more severe than in the saline group (*p* < 0.001) and continued for 8–10 days. The weight curve of the NM group reached a *plateau* at day 17 while control mice continued to actively gain weight. Mice, treated with different doses of AUY-922, showed visible improvements already during the first days of observation, and at day 23, the higher dose group (2 mg/kg 3×/week) demonstrated significant mass gain compared to mice instilled with NM and treated with saline (*p* < 0.01).

### 2.2. AUY-922 Blocks NM-Induced Alveolar Inflammation

**Figure 2:**
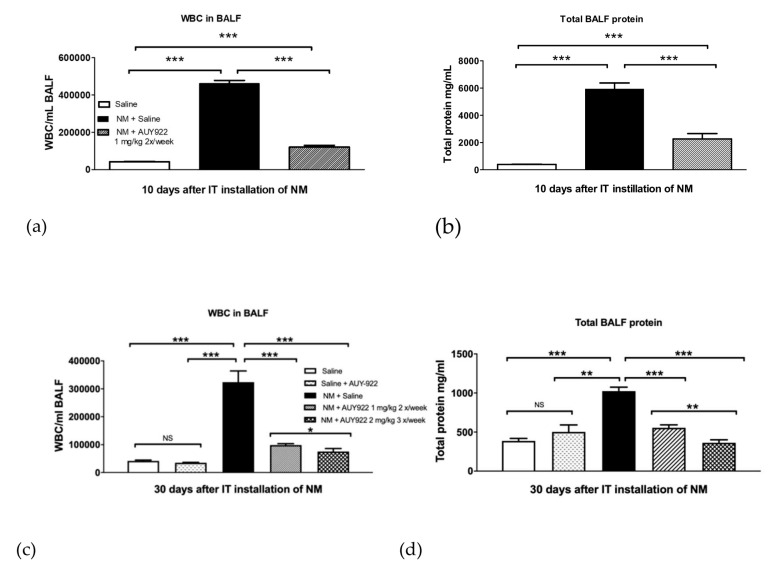
The HSP90 inhibitor, AUY-922, blocks NM-induced hypercellularity and increases total protein levels in bronchoalveolar lavage fluid (BALF). Mice received intratracheally 0.625 mg/kg NM or saline on day 0 and were treated with AUY-922 or saline for 10 (**a**,**b**) or 30 (**c**,**d**) days. Means ± SEM; ***: *p* < 0.001, **: *p* < 0.01, NS: not significant with ANOVA and Turkey’s. *n* = 6 mice per group.

As we previously published [18], NM elicits dramatic alveolar inflammation, which peaks at day 10 post-instillation and persists until day 30. Here, we analyzed white blood cells (WBC) and total protein concentration in bronchoalveolar lavage fluid (BALF) at 10- and 30-days post-exposure, in all groups. Mice instilled with 0.625 mg/kg NM and treated intraperitoneally with AUY-922 1 mg/kg, 2 times per week, showed significantly lower WBC levels already at 10 days when compared to controls (*p* < 0.001) (Figure 2a). After 30 days, this effect was sustained (*p* < 0.001). At the higher dose (2 mg/kg 3×/week), AUY-922 drastically diminished WBC concentration in BALF, when compared to the NM-treated mice (*p* < 0.001), exhibiting an even stronger effect than that observed in mice treated with AUY-922 1mg/kg 2×/week (*p* < 0.05; Figure 2c).

AUY-922 also reduced total protein BALF levels at 10 days (*p* < 0.001), and at 30 days post-exposure in both dosages (*p* < 0.001; *p* < 0.001) when compared to NM-instilled mice treated with saline (Figure 2b,d). Furthermore, the higher dose of AUY-922 showed a better effect than the lower one in reducing BALF protein concentration (*p* < 0.01). By itself, AUY-922 had no effect in vascular permeability and leucocyte migration.

### 2.3. AUY-922 Blocks NM-Induced Pulmonary Fibrosis

**Figure 3:**
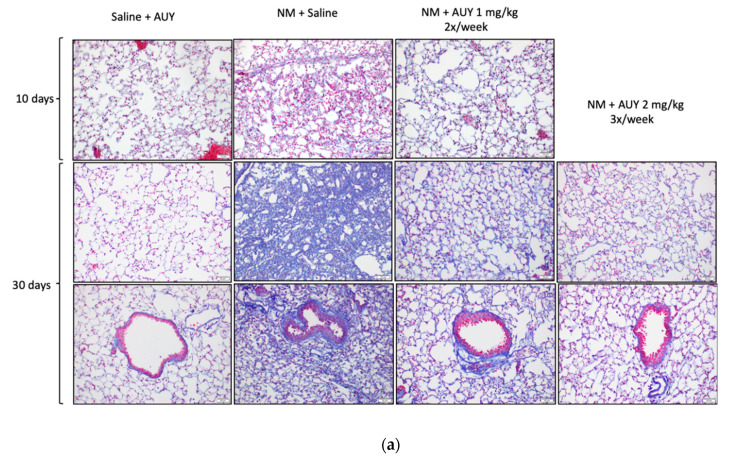

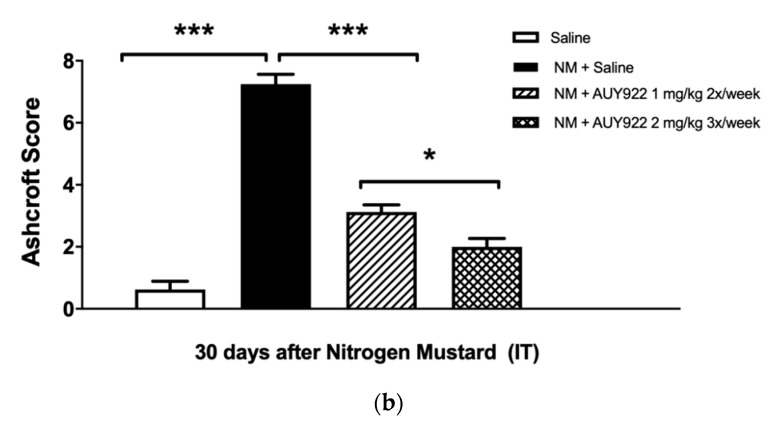
Histological analysis of lung injury after intratracheal administration of 0.625 mg/kg NM or saline followed by treatment, starting 24 h later, with either saline or the HSP90 inhibitor AUY-922 (1 mg/kg 2×/week or 2 mg/kg 3×/week i.p.) for 10 or 30 days. Masson’s trichrome staining of lung sections obtained 10 or 30 days after instillation (**a**). Formation of collagen filaments and significant increases in peribronchial and perivascular collagen were visible after instillation of NM at day 30. Mice treated with 1 mg/kg AUY-922 2×/week exhibited significantly less collagen deposition. Mice treated with 2 mg/kg AUY-922 3×/week showed only minor histological alterations. Original magnification: 20×, scale bars: 50 μm. Ashcroft score of lung fibrosis (**b**). Means ± SD; *n* = 4 mice per group; *: *p* < 0.05, ***: *p* < 0.001, with ANOVA and Tukey’s.

Fixed lung sections were stained with Masson’s trichrome stain to visualize lung architectural changes and estimate overall collagen deposition. At 10 days after NM instillation, an inflammatory process characterized by alveolar exudate swelling, alveolar deformation and leucocyte recruitment was observed (Figure 3a). Mice receiving AUY-922 showed fewer WBC in the alveolar space than controls, but other inflammatory signs, such as exudate and increased alveolar thickness persisted. Increased parenchymal, peribronchial and perivascular collagen deposition and large areas with fibrous obliteration were observed at day 30 in mice treated with 0.625 mg/kg NM. Moreover, increased number of macrophages were observed in the alveolar space and parenchymal tissue of mice instilled with NM. However, mice treated 2 times per week with 1 mg/kg AUY-922 displayed conserved parenchymal architecture and a lower collagen deposition, as reflected in the Ashcroft score, when compared to the NM-treated mice (*p* < 0.001; Figure 3b). Mice treated with the higher AUY-922 dose showed minor histological alterations, such as a slight increase in alveolar wall thickening without clear damage to lung architecture, and a subsequent lower Ashcroft score than the lower dose group (*p* < 0.05). Mice instilled with saline and treated with AUY-922 did not show any histological changes, in parenchymal morphology, Ashcroft score or peribronchial collagen deposition, when compared to saline-treated mice.

### 2.4. AUY-922 Prevents NM-Induced Overexpression of Extracellular Matrix Proteins

**Figure 4:**
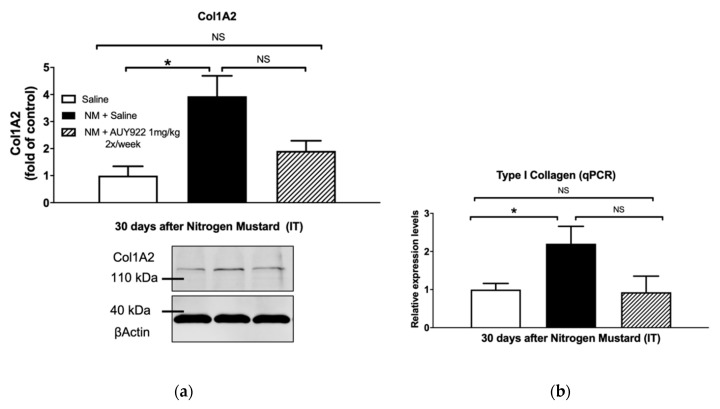

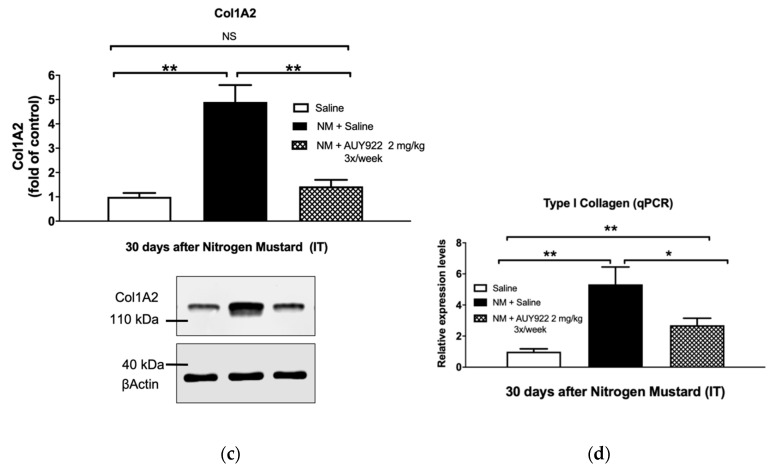
The HSP90 inhibitor, AUY-922, blocks the NM-induced collagen production. Total collagen protein and mRNA in lung tissue homogenates was determined by Western blotting (**a**,**c**) and real time qPCR (**b**,**d**), respectively, as described in the Methods. Mice received 0.625 mg/kg of NM or saline (2 µL/g body weight, intratracheally) on day 0. They then received AUY-922 (1 mg/kg, 2×/week (**a**,**b**) or 2 mg/kg, 3×/week (**c**,**d**) intraperitoneally) for 30 days, starting 24 h after NM. Means ± SEM; *n* = 4 mice per group; *: *p* < 0.05, **: *p* < 0.01, NS: not significant with ANOVA and Turkey’s.

**Figure 5:**
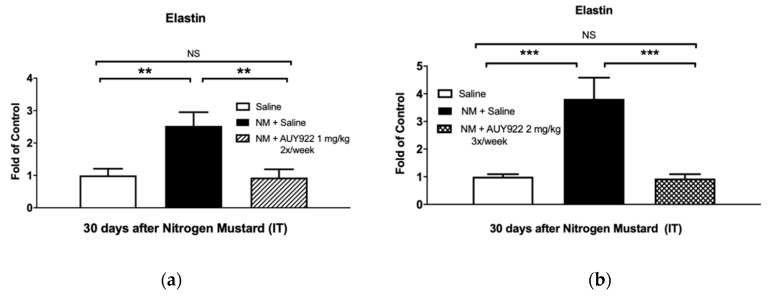
The HSP90 inhibitor, AUY-922, blocks the NM-induced elastin production. Total elastin mRNA levels in lung tissue homogenates was determined by real time qPCR, as described in Methods. Mice received 0.625 mg/kg of NM or saline (2 µL/g body weight, intratracheally) on day 0. They then received AUY-922 (1 mg/kg, 2×/week (**a**) or 2 mg/kg, 3×/week (**b**) intraperitoneally) for 30 days, starting 24 h after NM. Means ± SEM; *n* = 4 mice per group; **: *p* < 0.01, ***: *p* < 0.001, NS: not significant, with ANOVA and Tukey’s.

**Figure 6:**
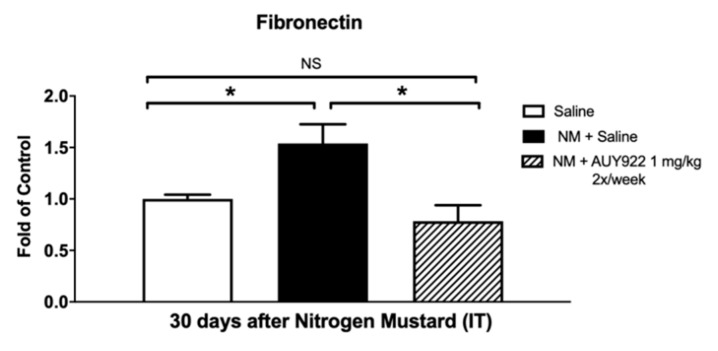
The HSP90 inhibitor, AUY-922, blocks the NM-induced fibronectin production. Total fibronectin mRNA in lung tissue homogenates was determined by real time qPCR, as described in the Methods. Mice received 0.625 mg/kg NM or saline (2 µL/g body weight, intratracheally) on day 0. They then received AUY-922 (1 mg/kg, 2×/week intraperitoneally) for 30 days, starting 24 h after NM. Means ± SEM; *n* = 4 mice per group; *: *p* < 0.05, NS: not significant with ANOVA and Tukey’s.

We analyzed collagen type I, elastin and fibronectin, key components of the lung extracellular matrix, involved in the maintenance of lung morphology and function. NM exposure in mice has been previously related to increased expression of collagen type I, fibronectin and elastin [18]. Treatment with 1 mg/kg 2×/week AUY-922 did not statistically reduce collagen type I deposition and mRNA expression (Figure 4a,b), however when AUY-922 was employed at the higher dose and frequency (2 mg/kg 3×/week) a significant reduction was observed in collagen, either analyzed by Western blotting (*p* < 0.01) or real-time qPCR (*p* < 0.05) (Figure 4c,d). The lower dose of AUY-922 was able to reduce the expression of both elastin (*p* < 0.01) and fibronectin (*p* < 0.05) when compared to NM-instilled mice treated with saline (Figure 5 and Figure 6).

### 2.5. AUY-922 Inhibits the Activation (Phosphorylation) of Pro-Fibrotic Biomarkers, ERK and HSP90

**Figure 7:**
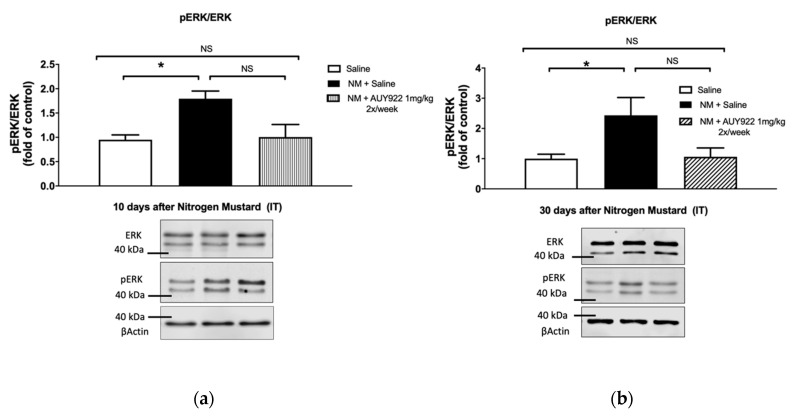

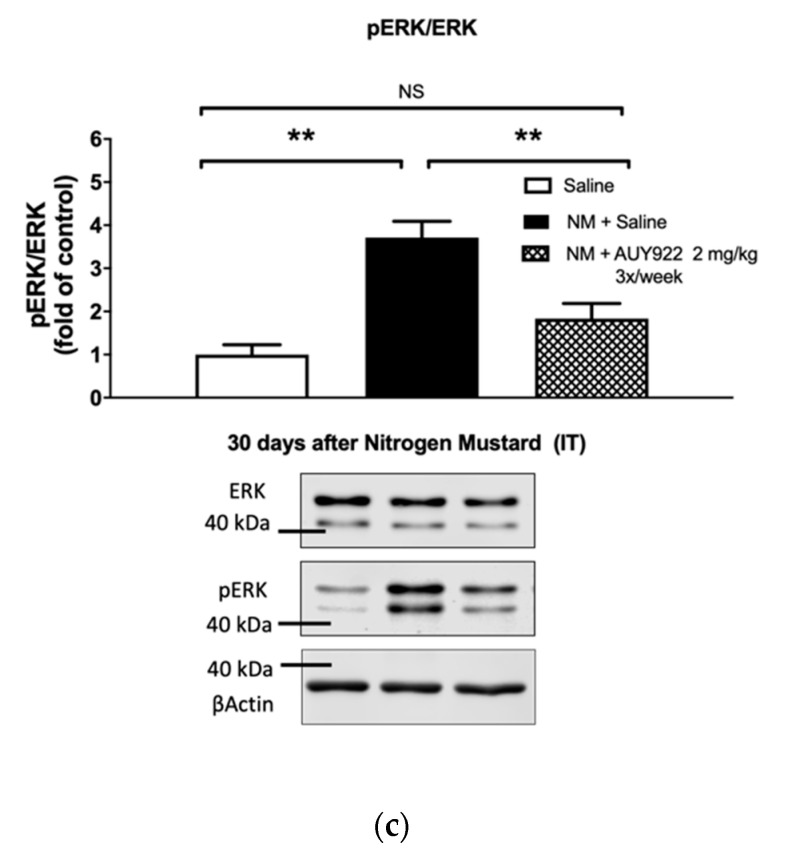
The HSP90 inhibitor, AUY-922, blocks the NM-induced increase in ERK activation (ERK phosphorylation, p-ERK) in mouse lung homogenates, analyzed by Western blotting. Mice received 0.625 mg/kg NM or saline (2 µL/g body weight, intratracheally) on day 0. They then received AUY-922 (1 mg/kg, 2×/week or 2 mg/kg 3×/week intraperitoneally) for 10 (**a**) or 30 (**b**,**c**) days, starting 24 h after NM. Protein quantification is expressed as fold of control mice. Means ± SEM; *n* = 4 mice per group; *: *p* < 0.05, **: *p* < 0.01, NS: not significant with ANOVA and Turkey’s.

**Figure 8:**
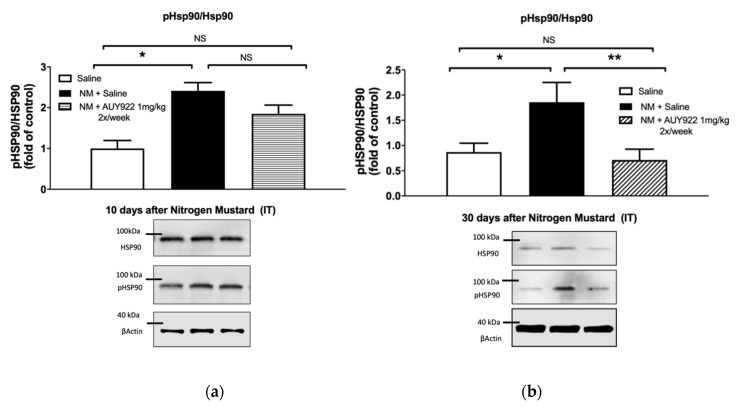

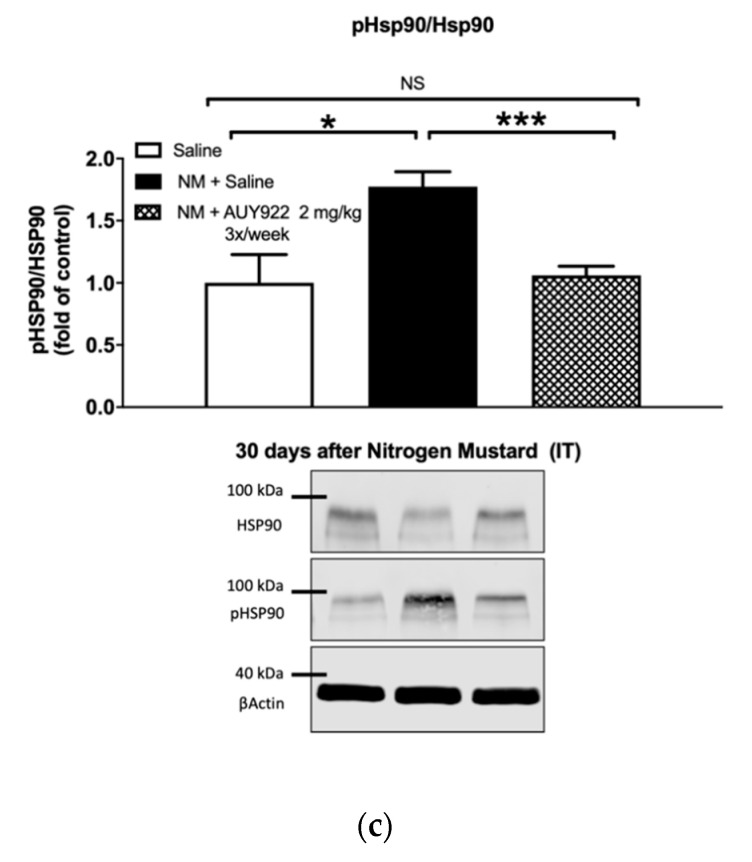
The HSP90 inhibitor, AUY-922, blocks the NM-induced increase in HSP90 activation (phosphorylation, p-HSP90) in mouse lung homogenates, analyzed by Western blotting. Mice received 0.625 mg/kg NM or saline (2 µL/g body weight, intratracheally) on day 0. They then received AUY-922 (1 mg/kg, 2×/week or 2 mg/kg 3×/week intraperitoneally) for 10 (**a**) or 30 (**b**,**c**) days, starting 24 h after NM. Protein quantification is expressed as fold of control mice. Means ± SEM; *n* = 4 mice per group; *: *p* < 0.05, **: *p* < 0.01, ***: *p* < 0.001, NS: not significant with ANOVA and Turkey’s.

We analyzed the ability of the HSP90 inhibitor AUY-922, to inhibit the expression of two pro-fibrotic signaling biomarkers: the activated (phosphorylated) form of extracellular signal-regulated kinase (MAPK/ERK) and of HSP90 (Figure 7 and Figure 8). As we previously reported, instillation of mechlorethamine hydrochloride increases the expression of p-ERK at 4, 10 and 30 days post-exposure [18]. NM also activates HSP90 (P-HSP90) by phosphorylating a tyrosine in position 300, at 30 days post-exposure. Administration of 1 mg/kg AUY-922 2 times per week did not significantly reduce p-ERK expression compared to the NM+saline group, at 10- and 30-days post-exposure (*p* > 0.05; *p* > 0.05; Figure 7a,b), even though the NM+AUY-922 group was statistically identical to the saline group. However, 2 mg/kg of AUY-922 injected 3 times per week significantly reduced p-ERK expression when compared to NM-instilled mice treated with saline (*p* < 0.01; Figure 7c). HP90 activation by NM was not affected by AUY-922 at day 10 post instillation, even though the NM+AUY-922 and saline groups were statistically similar. However, at 30 days post-exposure, both AUY-922 dosages successfully blocked HSP90 activation, when compared to NM-instilled mice treated with saline (lower dose: *p* < 0.01; higher dose: *p* < 0.001; Figure 8b,c). These data suggest that the phosphorylation of HSP90 is a continuous escalating process, which follows the progressive fibrotic process in the lungs.

### 2.6. AUY-922 Protects against NM-Induced Lung Dysfunction

**Figure 9:**
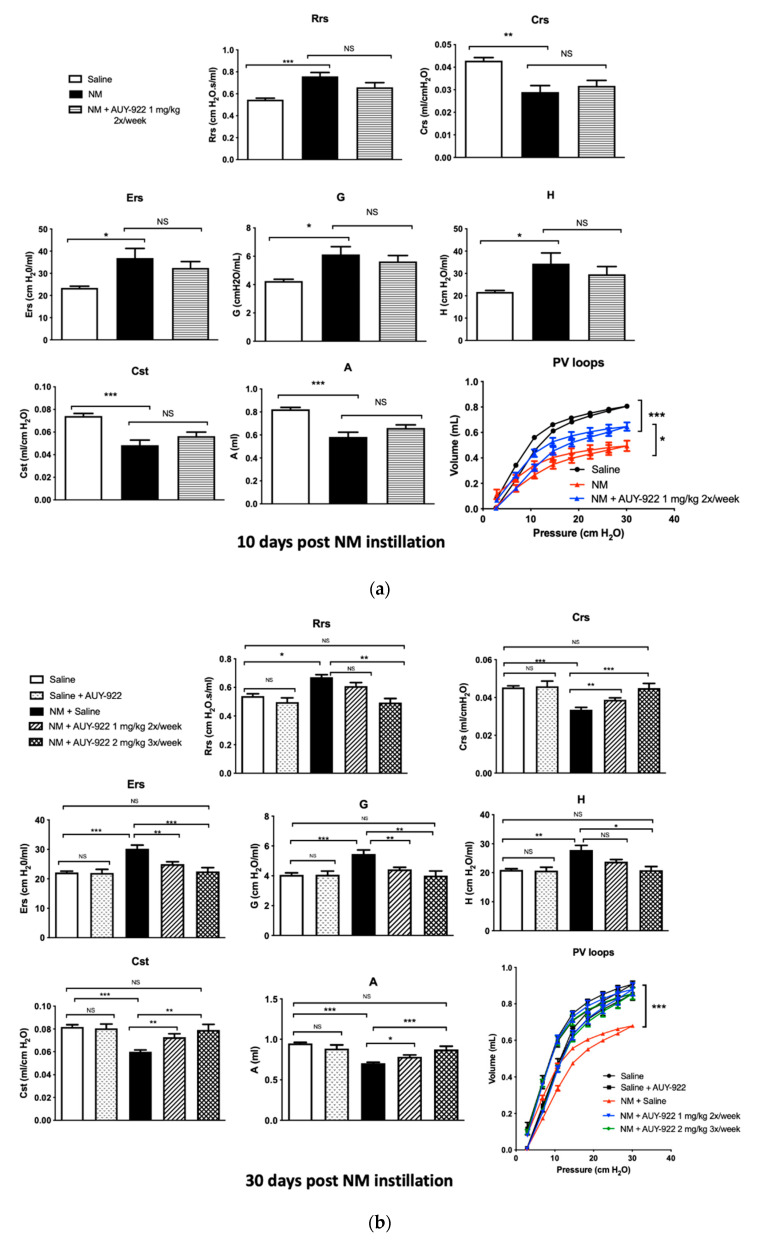
Effect of AUY-922 on NM-induced alterations in lung mechanics. Total respiratory system resistance (Rrs), total elastance (Ers), tissue elastance (H) and damping (G), increased, while respiratory system compliance (Crs), static compliance (Cst) and inspiratory capacity (A) decreased, compared to control mice in all groups, instilled with 0.625 mg/kg NM (**a**,**b**). The inhibitor had a significant impact on preventing a downward shift of pressure volume (PV) loops at 10 and 30 days after NM. Mice receiving AUY-922 for 30 days showed significant improvement in the values of all parameters studied, at both 1 mg/kg and 2 mg/kg dosages (B). Means ± SEM; *n* = 4 mice per group; *: *p* < 0.05, **: *p* < 0.01, ***: *p* < 0.001, NS: not significant, with ANOVA and Tukey’s.

Changes in lung mechanics were evident as early as 10 days after mechlorethamine hydrochloride instillation. Total respiratory system resistance (Rrs), elastance (Ers), tissue damping (G) and tissue elastance (H) increased significantly in NM-instilled mice at day 10 when compared to saline controls (*p* < 0.001; *p* < 0.05; *p* < 0.05 and *p* < 0.05, respectively). Similarly, 10 days after NM-instillation reduced values of respiratory system compliance (Crs), static compliance (Cst) and total lung capacity (A) were observed (*p* < 0.01; *p* < 0.001; *p* < 0.001, respectively). Treatment with AUY-922 1 mg/kg twice a week for 10 days did not result in significant improvements (Figure 9a). However, when the AUY-922 was administered for 30 days, it completely prevented the loss of compliance and lung stiffness at both dosages (Figure 9b). The study of lung function by FlexiVent also revealed that pressure–volume (PV) loops of NM-instilled mice treated with saline exhibited a characteristic downward shift reflecting stiffer lungs, while PV loops of mice treated with AUY-922 displayed a significantly lesser shift at 10 days (*p* < 0.05) and at 30 days reached physiological values compatible with controls (*p* < 0.001). AUY-922 at both dosages improved lung function after NM-exposure, however when employed at 2 mg/Kg 3×/week, it showed a stronger effect restoring respiratory systemic resistance (Rrs; *p* < 0.01). Taken together these data suggest that AUY-922 restores lung function in a time- and dose-dependent manner.

## 3. Discussion

In this study we used the HSP90 inhibitor, AUY-922 to combat the adverse effects associated with intratracheal exposure to nitrogen mustard. Currently, HSP90 inhibitors alone or in combination with chemotherapy drugs are in clinical trials for different types of cancer [19]. AUY-922 is an example of the second generation of HSP90 inhibitors, characterized by lower toxicity and higher therapeutic efficacy, already in phase 2 clinical trials as a potential anticancer drug. Similar to other HSP90 inhibitors that entered clinical evaluation, AUY-922 targets the ATP nucleotide-binding pocket located in the N-terminal domain of the HSP90 molecule, which is critical for its chaperone activity [20]. We selected this inhibitor based on its already proven pharmacological activity and clinical safety profile.

In bleomycin-induced pulmonary fibrosis models, mice demonstrated marked weight loss in the first week after exposure without showing a clear trend of recovery when compared to controls [21]. We observed a similar dynamic after the instillation of 0.625 mg/kg NM [18]. Here, we show how the administration of AUY-922 2 mg/kg 3 times per week significantly reduced the rate of weight loss compared to mice instilled with NM and treated with saline (Figure 1). Models of bleomycin-induced pulmonary fibrosis showed a persistent inflammatory process that lasts for at least 28 days post-exposure [22,23,24] and NM-instilled mice, similarly, displayed a strong alveolar inflammation that persists for at least 30 days [18]. We hypothesized that the inhibition of HSP90 by AUY-922 could suppress the persistent inflammatory process in the lung and prevent the development of NM-induced pulmonary fibrosis. To that end, we analyzed the WBC and total protein concentrations in BALF, which indicate alveolar inflammation, immune cell migration and, indirectly, alveolar-capillary hyper-permeability. Indeed, AUY-922 effectively suppressed both the alveolar inflammatory hypercellularity and proteinosis, as early as 10 days post-instillation and lasting at least until 30 days after NM. Furthermore, this was achieved at both dosage and frequency regimens of the drug. HSP90 inhibition, in fact, has been shown to exert a beneficial effect in attenuating acute lung inflammation [16,25] and different researchers have indicated how HSP90 plays a major role in endothelial barrier function, through its interaction with endothelial nitric oxide synthase (eNOS) [26,27]. Inhibitors of HSP90 restore endothelial barrier function in either LPS- or TGF-β1-induced barrier dysfunction models through inhibiting HSP90 interaction with activin receptor-like kinase 1-5 (ALK1 and ALK5) [28,29]. Here, treatment with AUY-922 and inhibition of HSP90 in NM-instilled mice, reduced WBC migration and BALF total protein levels, suggesting its anti-inflammatory action in the lung and, in part, its modulatory role on the endothelium.

The deposition of collagen and extra cellular matrix (ECM) proteins are responsible for the loss of parenchymal elasticity, reduced compliance and the changes in lung dynamics [30,31]. Increased levels of TGF-β are considered to be the causable factor for the fibrotic derangements in pulmonary fibrosis [32] but also in other fibrotic diseases [33,34,35]. TGF-β intracellular signaling represents a complex network that involves several kinases classified in two main cascades: Smad- and non-Smad-dependent [36]. HSP90 is involved in both as many of its client proteins belong to the non-Smad dependent pathway and, at the same time, HSP90 is able to modulate Smad localization [37]. The HSP90 inhibitor, 17-allylamino-17-demethoxygeldanamycin (17AAG), attenuates renal fibrosis through inhibition of Smad phosphorylation [38]. Gambogic acid, another HSP90 inhibitor, successfully reduced liver fibrosis through degradation of the HSP90 client proteins PI3K/AKT and the MAPK signaling pathways in both in vitro and in vivo studies [39]. In addition, disruption of the TGF-βRI-HSP90 complex by engineered HSP90 inhibitors reduced the myocardial collagen deposition induced by Angiotensin II, in vivo [40].

Previous studies have demonstrated that fibrotic changes in the lungs are evident at 3 and 7 days post NM exposure and, at 28 days, prominent collagen deposits are noted in the subpleural regions of the lung [5]. We previously observed visible perivascular and peribronchial collagen deposition, substitution of lung parenchyma with a fibrotic scar and narrowing of bronchial and alveolar spaces by the surrounding collagen. TGF-β also showed significant rise at 10 and 30 days after NM instillation [18]. Accordingly, in this study, mice exposed to NM exhibited increased collagen levels as analyzed by Masson’s Trichrome histological staining, real-time PCR and Western blotting. However, at the higher dosage, AUY-922 successfully reduced the Ashcroft score, collagen deposition and expression levels, when compared to mice exposed to NM and treated with saline. High levels of other ECM proteins, such as elastin and fibronectin have been well documented in different murine models of chemically-induced fibrosis, including the NM-induced model [18,31,41]. Here, we provide evidence that treatment with 2 mg/kg AUY-922, three times per week for 30 days reduces the mRNA expression levels of elastin and fibronectin to those of healthy controls.

HSP90 plays a critical role in regulating fibroblast activation in pulmonary fibrosis and represents a promising target for innovative therapeutic approaches [13]. Pharmacological inhibition of HSP90 ATPase activity was sufficient to reduce bleomycin-induced dermal fibrosis in Tsk-1 mice [13]. Furthermore, 17-AAG, a first generation HSP90 inhibitor, attenuates the development of pulmonary fibrosis in bleomycin-treated mice [16]. This inhibitor also blocked the interaction between the Hsp90 and TGF-β type II receptor, thereby preventing the development of renal fibrosis [38]. AUY-922, a second generation of HSP90 inhibitors, has shown higher potency and lower toxicity in clinical trials [42,43,44]. To investigate how AUY-922 interferes within intracellular signaling pathways and exerts its anti-fibrotic effects in the lung, we studied the expression levels of p-ERK and p-HSP90. We observed an increased expression of p-ERK and p-HSP90 10 days after NM exposure, which lasted up to 30 days. Activation of ERK has been involved in the pathogenesis and progression of different types of diseases [45]. Additionally, HSP90 regulates many proteins and it is critically involved in important signaling pathways including the ERK signaling pathway [46]. The MAPK/ERK pathway controls or modulates several critical cellular processes including growth, cell proliferation, differentiation, motility, the response to different cellular stressors, as well as survival and apoptosis [47]. TGF-β1 has been reported to operate through non-Smad dependent pathway involving small GTPases (RhoA and Cdc42), MAPK (ERK1/2, JNK, p38) and ERK1/2, which was reported to activate type I collagen and CTGF synthesis [48]. In this study we show that 30-days treatment with AUY-922 at the dose of 2 mg/kg, 3 times per week, significantly lowers the expression levels of activated ERK. In addition, we found that the administration of 1 mg/kg AUY-922 twice per week already decreased p-HSP90 levels at 30 days post-exposure.

The instillation of NM in mice provoked drastic changes in lung dynamics such as elevated Ers, Rrs, Rn, G and H and decreased Crs, Cst and A. Furthermore, NM-instillation modified pressure–volume loops, consistent with a restrictive pattern of pulmonary fibrosis. As we previously published, NM-exposure does not affect Newtonian resistance (Rn) and, differently from sulfur mustard exposure, provokes a minimal obstruction of the airways [18,49]. Interestingly, the restrictive pattern observed, appeared at day 10 and was present at least till day 30, suggesting a non-reversible alteration in lung structure following NM exposure. Mice exposed to NM and treated with AUY-922 (2 mg/kg 3×/week) for 30 days successfully restored systemic resistance, elastance, static and dynamic compliance, improved lung capacity and corrected the downward shift of PV curves. The beneficial effects of AUY-922 in preventing changes of the respiratory system resistance (Rrs) and elastance (Ers) were demonstrated also in a hydrochloric acid-induced model of pulmonary fibrosis in mice, which however require a lower dose and length of treatment [50].

In this study, AUY-922 administration in control mice (saline) did not show significant alterations in inflammation, vascular permeability, collagen expression, phosphorylation of either ERK or HSP90 and histology, suggesting a safe profile in healthy subjects.

Thus, we confirmed that AUY-922 prevents the development of NM-induced pulmonary fibrosis by modulating the levels of pro-fibrotic markers, reducing collagen expression and deposition and maintaining normal lung dynamics. The suppression of p-HSP90 by low doses of AUY-922 interferes critically within the development of pulmonary fibrosis and may represent a new approach for victims exposed to nitrogen mustard gases.

## 4. Material and Methods

### 4.1. Materials

Mechlorethamine hydrochloride 98%, red protein G affinity gel beads, RIPA buffer and protease inhibitor cocktail were obtained from Sigma-Aldrich Corporation (St. Louis, MO, USA). The HSP90 Inhibitor AUY-922 (NVP-AUY922, 99.1% purity) was purchased from Selleck Chemicals (Houston, TX, USA). Socumb (pentobarbital) United States Pharmacopeia (USP) grade, Anased (xylazine) USP grade and Ketaset (ketamine) USP grade were supplied by Henry Schein Animal Health (Pittsburg, PA, USA). 10% formaldehyde was purchased from Thermo Fisher Scientific (Waltham, MA, USA), EDTA and Western blot membranes from GE Healthcare (Chicago, IL, USA), TRIzol^®^ and SuperScript™ IV VILO Reverse transcription Kit from Invitrogen (Carlsbad, CA, USA), RNeasy Mini Kit from QIAGEN (Germantown, MD, USA) and SYBR Green Master Mix from Applied Biosystems (Carlsbad, CA, USA). All primers used for real time quantitative PCR were purchased from Integrated DNA Technologies, Inc. (Coralville, IA, USA). All antibodies used in Western blots and immunoprecipitation have published immunospecificity data available online. For antibodies used in Western blots, rabbit total and phosphorylated Erk1/2 were obtained from Cell Signaling Technology, Inc. (Danvers, MA, USA), mouse monoclonal anti-β-actin from Sigma-Aldrich Corporation, and IRDye 800CW Goat anti-rabbit and IRDye 680RD Goat anti-mouse from LI-COR Biosciences (Lincoln, NE, USA). For antibodies used in immunoprecipitation, mouse anti-phosphotyrosine was from Invitrogen, and mouse anti-Hsp90 from BD Transduction Laboratories (Franklin Lakes, NJ, USA).

For preparation of SDS-PAGE: ProtoGel (30% acrylamide mix) and TEMED were from National Diagnostics (Atlanta, GA, USA), Tris-HCl buffer from Teknova (Hollister, CA, USA), 10% SDS and ammonium persulfate from Thermo Fisher Scientific, and protein dual color standards and Tricine sample buffer were purchased from Bio-Rad Laboratories.

### 4.2. Animals and Treatment Groups

All animal studies were approved by the Old Dominion University IACUC and adhere to the principles of animal experimentation as published by the American Physiological Society. Male pathogen-free C57Bl/6J (Jackson Laboratories, Bar Harbor, ME, USA) mice (8–10 weeks old, 24–28 g weight) were randomly divided into eight treatment groups:

1. Saline 30 days group: mice received 2 μL/g body weight saline intratracheally (i.t.) and were then treated with 0.1 mL saline intraperitoneally (i.p.), 2 times/week for 30 days (*n*  =  12 mice). All analyses were performed at 30 days post i.t. saline exposure.
2. Saline + AUY-922 30 days group: mice received 2 μL/g body weight saline i.t. and were then treated with 1 mg/kg AUY-922 i.p. for 30 days (*n*  =  12 mice). All analyses were performed at 30 days post i.t. saline exposure.
3. Saline 10 days group: mice received 2 μL/g body weight saline, i.t. and were treated with 0.1 mL saline (i.p.) 2 times per week for 10 days (*n*  =  12 mice). All analyses were performed at 10 days post saline i.t. exposure.
4. NM + Saline 10 days group: mice were exposed to 0.625 mg/kg body weight NM (i.t.) and were treated with 0.1 mL saline (i.p.) 2 times per week for 10 days (*n*  =  12 mice). All analyses were performed at 10 days post NM exposure.
5. NM + AUY-922 10 days group: mice were exposed to 0.625 mg/kg body weight NM (i.t.) and were treated with AUY-922 1mg/kg (i.p.) 2 times per week for 10 days (*n*  =  12 mice). All analyses were performed at 10 days post NM exposure.
6. NM + Saline 30 days group: mice were exposed to 0.625 mg/kg body weight NM (i.t.) and were treated with saline (i.p.) for 30 days (*n*  =  12 mice). All analyses were performed at 30 days post NM exposure.
7. NM + AUY 1 mg/kg 30 days group: mice were exposed to 0.625 mg/kg body weight NM (i.t.) and were treated with AUY-922 1 mg/kg (i.p.) 2 times/week for 30 days (*n*  =  12 mice) All analyses were performed at 30 days post NM exposure.
8. NM + AUY 2 mg/kg 30 days group: mice were exposed to 0.625 mg/kg body weight NM (i.t.) and were treated with AUY-922 2 mg/kg (i.p.) 3 times/week for 30 days (*n*  =  12 mice) All analyses were performed at 30 days post NM exposure.

A stock solution of AUY-922 in 10% DMSO was prepared and stored at −20 °C for up to 30 days. Mice were treated with freshly prepared solution of AUY-922 in pure sterile saline (1 or 2 mg/kg in a volume of 0.1 mL) from the stock prior to each intraperitoneal injection.

For i.t. administration of mechlorethamine or saline, mice were first anesthetized with i.p. injections of xylazine (6 mg/kg) and ketamine (60 mg/kg). An i.p. bolus of sterile normal saline (10 μL/g) was then given as pre-emptive fluid resuscitation. A small neck skin incision (1 cm) and separation of the salivary glands was made to visualize the trachea. Mice were positioned vertically, a fine (20 G) plastic catheter was advanced into the trachea through the mouth (2 cm) and its position was visually verified. Then, freshly prepared mechlorethamine hydrochloride or sterile saline solution was introduced (2 μL/g body weight) and flushed with 100 μL air. The catheter was withdrawn, the skin incision was closed by surgical adhesive and animals were placed in the ventral position under supplemental oxygen (slowly weaned from 100 to 21% O_2_) and observed for the next four hours for signs of respiratory distress. Mice were later returned to the home-cages and weighed and monitored daily for abnormal physical appearances. The HSP90 inhibitor, AUY-922 was administered twice or three times a week starting 24 h after NM instillation. After 10 or 30 days, mice were euthanized and bronchoalveolar lavage fluid and whole lung tissue were collected for analysis. Using this model, we achieved 86% survival rate for mice exposed to mechlorethamine hydrochloride and a 100% survival for mice treated with saline.

### 4.3. Lung Mechanics Measurements

Mice from all groups were anesthetized with pentobarbital (90 mg/kg, i.p.), tracheostomized with a metal 1.2 mm (internal diameter) cannula and connected to a FlexiVent small animal ventilator (Scireq Inc., Montreal, QC, Canada). Ventilation was performed at a tidal volume of 10 mL/kg and respiratory rate of 150/min. A 15-min stabilization period was allowed before any measurements began. Two sets of studies were done, each including mice from the aforementioned treatment groups. In one set, we first performed the Snapshot-150 maneuver, which is a brief (1.25 sec) single frequency forced oscillation at a tidal volume of 10 mL/kg and respiratory rate of 150/min. Volume and pressure signals were recorded and fit into a single compartment model to derive total resistance (Rrs) and elastance (Ers), reflecting the behavior of the entire respiratory system (peripheral and conducting airways, chest wall and parenchyma). After a second 15-min rest period, the Quick Prime maneuver was performed, consisting of forced oscillations at a series of frequencies that allow calculations of impedance at each frequency. Data are then fit into a constant phase model to estimate Newtonian resistance (Rn) and tissue damping (G) values, the former reflecting resistance of the large, conducting airways and the latter reflecting mostly parenchymal and peripheral airway contributions. In the second group of studies (different set of mice), resting static compliance (Cst) and pressure-volume relationships (PV curves) were estimated by stepwise increasing airway pressure to 30 cm H_2_O and then reversing the process. Both parameters reflect the intrinsic elasticity of the lungs and are either reduced (Cst) or shifted to the right (PV curves) in fibrosis.

### 4.4. Histopathology and Lung Injury Scoring

Instantly after euthanasia, mice were positioned upright, the lungs were instilled and inflated with 10% formalin solution to a pressure of 15 cm H_2_O and then immersed in the same solution for at least 72 h prior to histology. After fixing, lung tissue samples were embedded in paraffin. For collagen staining, 5 μm thick sections were prepared from the paraffin blocks and stained with Masson’s trichrome. Twenty randomly selected fields from each slide were examined under 20× magnification. Slides were scored according to the Ashcroft score method [51] in order to estimate the severity of pulmonary fibrosis. A person blinded to the study protocol performed the scoring.

### 4.5. Bronchoalveolar Lavage Fluid (BALF) and White Blood Cell Count

Bronchoalveolar lavage fluid (BALF) was collected by instilling and withdrawing sterile 1× PBS (1 mL) via the tracheal cannula. The fluid was centrifuged at 2500× *g* for 10 min at 4 °C (Thermo Fisher Centrifuge 5417R) and the supernatant was collected and stored at −80 ˚C. The cell pellet was resuspended in 1 mL sterile PBS and the total number of white blood cells was counted using a hemocytometer.

### 4.6. Total Protein in BALF

BALF supernatant was prepared as described in the previous section and analyzed for total protein content, which was detected using the micro bicinchoninic acid (BCA) assay according to the manufacturer’s protocol.

### 4.7. Lung Tissue Collection

After euthanasia, the thorax was opened, blood was drained from the heart and the pulmonary circulation was flushed with sterile PBS containing EDTA. The lungs were dissected, frozen in liquid nitrogen and stored at −80 °C until analyzed.

### 4.8. Western Blot Analysis

Proteins in lung tissue homogenates were extracted from frozen lungs by sonication (50% amplitude, 3 times for 10 s) in ice-cold RIPA buffer with added protease inhibitor cocktail (100: 1). The protein lysates were gently mixed under agitation for 3 h at 4 °C, and then centrifuged twice at 14,000× *g* for 10 min. The supernatants were gathered, and total protein concentration was determined using the micro BCA assay. Equal amounts of proteins from all lysates were used for Western blot analysis. The samples were first mixed with tricine sample buffer 1:1, boiled for 5 min and then separated on a 10–12% polyacrylamide SDS gel by electrophoresis. Separated proteins were then transferred to a nitrocellulose membrane, incubated with the appropriate primary antibody, followed by incubation with the secondary antibody and detected by digital fluorescence imaging (LI-COR Odyssey CLx, Dallas, TX, USA). Beta-actin was used as loading control. ImageJ software v.1.8.0was used to perform densitometric quantification of the bands from the Western blot membranes (http://imagej.nih.gov/ij/; National Institutes of Health, Bethesda, MD, USA).

### 4.9. Immunoprecipitation Procedure

Protein lysates for immunoprecipitation were obtained following the procedure described in the previous section (Western blot analysis) and analyzed for total protein.

Immunoprecipitation protocol: The primary antibody (mouse anti-Hsp90) was added to the lysates (1:200) containing 1000 µg of protein and incubated overnight at 4 °C under gentle agitation using a rotary mixer (Thermo Scientific). The next day, red protein G affinity gel beads were added to the lysates and the suspensions were incubated for 3 h under rotary agitation at 4 °C. The lysate-bead suspensions were pelleted and then washed four times with RIPA lysis buffer. In the final step, the supernatant was removed from the suspension by centrifugation for 10 min at 4 °C and beads were mixed with tricine sample buffer (1:1). The proteins were separated from the beads by heat denaturation at 95 °C for 10 min. The protein samples were then subjected to Western blot analysis, the resulting protein bands were detected and densitometric quantification of the protein bands was performed as described above.

### 4.10. RNA Isolation and Quantitative Real-Time PCR (qPCR)

Total RNA was isolated from lung homogenates using TRIzol^®^ followed by a cleaning up step with QIAGEN RNeasy Mini Kit. The purified RNA was transcribed into cDNA using the SuperScript™ IV VILO Reverse transcription Kit and analyzed by real-time qPCR with SYBR Green Master Mix on a StepOne Real-Time PCR System (Applied Biosystems, Foster City, CA, USA). Results were evaluated using the standard curve method and expressed as the fold of control values. Beta-actin was used for the normalization of each mRNA expression level for all samples. Specifically designed primer pairs and qPCR conditions were applied to selectively determine the expression of mouse beta-actin as previously described.

### 4.11. Statistical Analysis

Statistical significance of differences among groups was determined by one- or two- way analysis of variance (ANOVA) followed by the Tukey post-hoc tests using Prism 8 (GraphPad Software, San Diego, CA, USA). Differences among groups were considered significant at *p* < 0.05.

## 5. Conclusions

In the present study, we demonstrated for the first time that AUY-922, a second-generation HSP90 inhibitor, prevented the development of chronic lung injury and pulmonary fibrosis in NM exposed mice, modulating lung inflammation, pro-fibrotic biomarkers and deposition of ECM proteins and collagen. Furthermore, our data show that a 30-day treatment with AUY-922 2 mg/kg 3×/week was safe in control mice and performed more effectively than the 1 mg/kg, 2×/week regimen in preventing NM-induced pulmonary fibrosis. Further studies are needed to define the most effective and safe treatment regimen in different animal models that could eventually be translated to humans [35,46].
